# Preventive Behavioral Responses to the 2015 Middle East Respiratory Syndrome Coronavirus Outbreak in Korea

**DOI:** 10.3390/ijerph16122161

**Published:** 2019-06-18

**Authors:** Won Mo Jang, Sanghyun Cho, Deok Hyun Jang, Un-Na Kim, Hyemin Jung, Jin Yong Lee, Sang Jun Eun

**Affiliations:** 1 Health Review and Assessment Committee, Health Insurance Review and Assessment Service, Wonju 26465, Korea; thomasjang.ph@gmail.com (W.M.J.); hyeminjung82@gmail.com (H.J.); 2 Department of Health Policy and Management, Seoul National University College of Medicine, Seoul 03080, Korea; jsanghun@gmail.com; 3 Research Analytics & Communication, Gallup Korea, Seoul 03167, Korea; dhjang@gallup.co.kr; 4 Bureau of Health Policy, Ministry of Health and Welfare, Sejong 30113, Korea; wickedbeam@naver.com; 5 Department of Public Health and Community Medicine, Seoul Metropolitan Government—Seoul National University Boramae Medical Center, Seoul 07061, Korea; 6 Department of Preventive Medicine, Chungnam National University College of Medicine, Daejeon 35015, Korea

**Keywords:** Middle East respiratory syndrome coronavirus, disease prevention and control, disease outbreaks, health survey

## Abstract

This study examined the public’s preventive behavioral responses during the 2015 Middle East respiratory syndrome coronavirus (MERS-CoV) outbreak in Korea and the influencing factors. Two cross-sectional telephone surveys were conducted by Gallup Korea using random digit dialing in June 2015 (*n* = 2004). The main outcome variables were nonpharmaceutical preventive measures (survey (1): Measures for reducing transmission (handwashing, face masks); and survey (2): Measures for avoiding contact with others). Multiple logistic regression was used to identify the factors influencing preventive behaviors. In survey (1), 60.3% of respondents reported more frequent handwashing and 15.5% reported wearing face masks at least once due to the MERS-CoV epidemic. In survey (2), 41–56% of respondents reported practicing avoidance measures. The concerned group was more likely to practice reducing transmission measures (odds ratio (OR) 4.5; 95% confidence interval (CI) 3.3–6.1) and avoidance measures (OR = 9.6; 95% CI, 6.4–14.4). The respondents who had low trust in president or ruling party had a higher practice rate of reducing transmission measures (OR = 1.7; 95% CI, 1.2–2.6) and avoidance measures (OR = 2.1; 95% CI, 1.2–3.5). Cooperative prevention measures need appropriated public concern based on effective risk communication.

## 1. Introduction

The Middle East Respiratory Syndrome (MERS) epidemic in Korea has had a huge impact on Korean society. Beginning on 20 May 2015, after a patient diagnosed with MERS returned from travelling to the Middle East, a total of 186 cases were confirmed, 38 deaths occurred, and 16,693 patients were quarantined [1]. As a result, South Korea became the country with the highest number of MERS cases, apart from Saudi Arabia [2]. In addition, after a person who travelled to China following close contact with a confirmed MERS patient was confirmed with MERS in China, the government and non-governmental organizations expressed concerns that these cases could turn into a pandemic [3,4,5].

To control the spread of an infectious disease such as MERS, there is a need for communication and close cooperation between the government and the people [5]. However, some inappropriate actions of the government in the early stages of the MERS outbreak contributed to the spread of this epidemic. The government did not disclose which hospitals were managing MERS patients in the early stages of the outbreak, which not only increased the fear of the public regarding MERS but also increased the incidence of secondary infections by super-spreaders—who transmit an infection to a significantly greater number of other individuals than average [6]. In addition—unlike the Centers for Disease Control and Prevention in the USA, which responds to epidemics with several alternative scenarios to be prepared for uncertainty—the Korean government responded to the MERS outbreak in an overly optimistic manner [7]. The government established a national epidemic plan based on the assumption that MERS has low infectivity and set the standard of close contact as more than one hour and within two meters. A study conducted after the MERS outbreak revealed that MERS can be transmitted through short-term contact, and that there is the possibility of airborne transmission in the hospital by super-spreaders [8,9,10].

The cooperation of the population is also necessary to end an epidemic. The World Health Organization (WHO) has stated that from the time of outbreak of an epidemic, it is necessary to engage the community through radio-based activities and to seek cooperation to ensure that the population is prepared to take precautions at the individual level [11]. Previous studies have shown that preventive measures that individuals can practice are effective in reducing disease infection rates. Reducing physical contact between individuals in the workplace was found to reduce the rate of influenza transmission by 23% [12], and handwashing was reported to reduce the spread of severe acute respiratory syndrome (SARS) [13].

During the MERS outbreak in 2015, the government recommended wearing face masks and handwashing as preventive measures against MERS. However, no study has been performed yet investigating to what extent these measures were practiced. Identifying the preventive behavioral responses of the public and understanding their influencing factors can serve as an evaluation tool for the government for risk communication during the MERS epidemic, as well as providing basic information for effective communication of risks during future infectious disease outbreaks. Therefore, this study aimed to investigate the practice rates of individual nonpharmaceutical preventive measures during the outbreak of MERS in 2015 and to identify the factors influencing their practice.

## 2. Materials and Methods

### 2.1. Subjects

This study used the results of two surveys conducted by Gallup Korea in June 2015 during the MERS outbreak. Gallup Korea, an affiliation of Gallup International, conducts weekly telephone surveys of adults older than 19 years. The first survey for this study was conducted between June 2nd and 4th, at a time when fear was spreading after 30 cases had been confirmed and the first death caused by MERS had occurred. The second survey was conducted between June 23 and 25 after the high incidence of MERS had decreased and the number of newly confirmed cases was roughly 1–2 per day. The surveys were conducted using mobile and landline random digit dialing in eight cities and provinces. Samples were selected using post-stratification including gender, age, and province. The total number of weighted cases in this survey equals the total number of unweighted cases at the national level. The weights were normalized in order to give proportions and ratio, however not for estimating number of subtotal populations. The survey was conducted by trained interviewers using computer assisted telephone interviewing (CATI). The first survey had 1004 respondents with a response rate of 15.5%; and the second survey had 1000 respondents with a 17.7% response rate.

### 2.2. Variables

#### 2.2.1. Nonpharmaceutical Preventive Behaviors

Measures aimed at preventing MERS practiced at the individual level were investigated by dividing them into reducing transmission behaviors (handwashing and wearing face masks) and risk avoidance behaviors (avoiding outdoor activities, public transportation, visits to healthcare facilities, and crowded places). The name “MERS” was present in all questions to elicit responses about behavioral changes related to MERS. The first survey assessed reducing transmission behaviors (June 2–4th) using the following questions:

(a) Do you wash your hands more often than usual because of MERS?
(b) Have you ever worn a face mask because of MERS?

The second survey assessed risk avoidance behaviors (June 23–25th) using the following questions:

(a) Did you reduce or avoid outdoor activities or attending meetings this week because of MERS?
(b) Did you reduce or avoid using public transportation such as the bus or the subway this week because of MERS?
(c) Did you reduce or avoid using healthcare facilities such as hospitals or public health centers this week because of MERS?
(d) Did you reduce or avoid visiting crowded markets, department stores, or large discount stores this week because of MERS?

All questions about nonpharmaceutical preventive behavior required yes/no responses.

#### 2.2.2. Personal Characteristics

Gender, age, occupation, perceived household economic status, residential area, political orientation, and anxiety level regarding MERS were also investigated to identify factors influencing MERS nonpharmaceutical preventive behaviors. Occupation was classified as either unemployed, farming and fishery, self-employed, blue-collar worker, white-collar worker, full-time housewife, or student. Perceived household economic status was classified into five levels as lower, low middle, middle, upper middle, upper. Respondents were classified as metropolitan residents or non-metropolitan residents and distinguished by whether they resided in an area where MERS had occurred or not. Political orientation was classified based on support for the president and the political parties. Support or lack of support for the president was assessed using the options of “approval”, “disapproval”, or “no opinion”, while support for the political parties was assessed based on alignment with the ruling party (Saenuri in 2015), with the opposition party, or by no opinion. Concerns regarding MERS were assessed using the question "How worried are you about MERS infection?". Responses were assessed on a four-point scale, with four points indicating “very worried” and one point indicating “not worried at all”. A response of 3–4 points was classified as “worried”, while a response of 1–2 points was classified as “not worried”.

The second survey investigated the respondents’ education levels and predictions regarding the MERS epidemic. Education level was classified as middle school graduate or below, high school graduate, university graduate, or postgraduate. Predictions for the MERS epidemic were evaluated using the question “Do you think the MERS epidemic will subside or spread within a few days?” and required the responses “controlled,” “uncontrolled,” or “no opinion”.

### 2.3. Analysis

Using the results of the telephone surveys, the practice rates of reducing transmission behaviors and risk avoidance behaviors were calculated according to respondent characteristics. Missing values of nonpharmaceutical preventive behaviors variables were 1% or less by personal characteristics variables. Missing values were dropped from both the descriptive analyses and logistic regression. Logistic regression analysis was performed to explore factors influencing nonpharmaceutical preventive behaviors. Using logistic regression analysis for each reducing transmission behavior and risk avoidance behavior, “*y* = 1” was used when one or more reducing transmission or risk avoidance behaviors were practiced, otherwise “*y* = 0” was used.

### 2.4. Ethical Considerations

This study was reviewed and approved by the Institutional Review Board (IRB) of Seoul Metropolitan Government–Seoul National University Boramae Medical Center (IRB No. 20190515/07 - 2019 - 11/062). The need for informed consent was waived by the board.

## 3. Results

Table 1 shows the practice rates of reducing transmission behaviors according to respondent characteristics. Fifteen percent of all respondents reported wearing face masks because of MERS, and 60% reported that they washed their hands more often than usual because of MERS. Female subjects had higher practice rates of reducing transmission behaviors than male subjects. While practice rates of wearing face masks tended to be more frequent in the lower age group of 19–39 years, practice rates of handwashing were highest among those aged 40–49 years. With respect to occupation, practice rates of reducing transmission behaviors among white-collar workers, housewives, and students were higher than those among other occupations. Farmers and fishermen had the lowest practice rates of reducing transmission behaviors, and none of them reported wearing face masks because of MERS. There was no definitive trend observed with respect to perceived household economic status. Practice rates of reducing transmission behaviors were high in areas affected by MERS and in metropolitan areas. Meanwhile, practice rates of reducing transmission behaviors were high in the groups that did not support the president. Those who did not stand by the ruling party practiced more reducing transmission measures than those who did. In addition, the group concerned about MERS infection showed high practice rates of reducing transmission behaviors. People in this group were 3 times more likely to wear face masks and twice as likely to wash their hands compared with the group that was not worried about infection.

Table 2 shows the practice rates of risk avoidance behaviors according to respondent characteristics. Approximately 41–56% of respondents practiced risk avoidance behaviors. The most frequently practiced avoidance behavior was to avoid visiting hospitals and other medical institutions, while the least frequently practiced behavior was to avoid using public transportation. Risk avoidance behaviors were most commonly practiced by females, with the highest rate among those aged 30–39 years. The higher the education level, the higher the practice rate was. Housewives and white-collar workers had high practice rates; while unemployed, blue-collar and self-employed workers had low practice rates. There was no definitive trend observed with respect to perceived household economic status. There were no differences in reducing transmission measures between MERS-affected areas and non-affected areas, as well as between metropolitan and non-metropolitan areas. The group that did not indicate support for the president and the ruling party had high practice rates of risk avoidance behaviors. In addition, perception of MERS led to different risk avoidance behaviors. Risk avoidance behaviors were more commonly practiced among those who were worried about MERS infection as compared with the non-worried group, worried people were also twice as likely to practice all reducing transmission measures. The group more likely to predict that MERS would spread also had high rates of risk avoidance behaviors.

**Figure 1:**
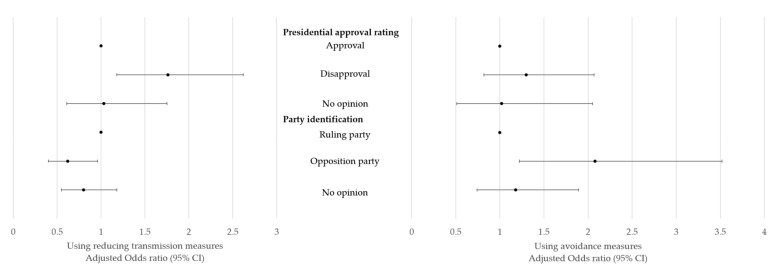
Adjusted odds ratios of nonpharmaceutical preventive behaviors according to political orientation.

Table 3 shows the factors associated with nonpharmaceutical preventive behaviors for MERS. The strongest associated characteristic related to both reducing transmission behaviors and risk avoidance behaviors was concern about MERS infection. Compared with the non-worried group, groups that were worried about MERS infection were 4.5 times more likely to practice reducing transmission behaviors and 9.6 times more likely to practice risk avoidance behaviors. Female participants were more likely to engage in nonpharmaceutical preventive behaviors than male participants were (odds ratio (OR), 1.5–1.7), while other demographic and socioeconomic characteristics such as age, education level, perceived household economic status, and area of residence were not significantly associated with preventive behaviors. Figure 1 shows the difference in practice rates of measures by political affiliations. The group that indicated support for the president had high practice rates of reducing transmission behaviors (OR, 1.8). The group that did not support the ruling party had low rates of reducing transmission behaviors (OR, 0.6) and high rates of risk avoidance behaviors (OR, 2.1). The rate of avoidance behaviors was found to not be significant, although it was found to be high among groups that predicted that MERS would spread (OR, 1.4).

## 4. Discussion

This study found that about half of respondents practiced preventive behaviors and showed different preventive behavioral responses. Previous studies that investigated preventive behaviors during prevalent infectious diseases also showed that the practice rates of various preventive behaviors differed and that the practice rates of clear and practical preventive behaviors were high [14,15]. According to a survey on preventive behaviors in the early stages of the influenza A virus subtype H1N1 outbreaks in 2009, there was a greater proportion of participants who washed their hands more often or avoided people with cold symptoms (55–67%) than of those who avoided crowded places, avoided contact with certain races, or cancelled their travel plans (13–27%) [14]. In a study on preventive behaviors during the SARS epidemic in 2003, practice rates of hygienic behaviors such as handwashing, wearing face masks, and household disinfection were also high at 65–87%; while rates of avoidance of certain places such as markets or hospitals as well as using public transportation were low at 24–75% [15]. As this study reported a low rate of wearing face masks, a behavioral characteristic similar to handwashing, further studies on the factors prompting face mask use are needed.

Despite the high level of public concern regarding infection during the 2015 MERS epidemic (52–67%) compared with the SARS epidemic in Hong Kong in 2003 (9–48%), a lower preventive behavioral response rate of 15–60% was seen with the MERS outbreak, as compared with the rate of 24–87% with the SARS epidemic [15]. This indicates that measures should be taken to increase preventive behaviors for infectious diseases. It is desirable to educate the population regarding health and preventive behaviors for infectious diseases regularly at schools and public institutions. Knowledge of the effectiveness of preventive behaviors increases the practice of such behaviors [16,17]. In addition, education would not only promote a positive perception of preventive behaviors but also improve the quality of preventive behaviors [18,19].

According to a multiple logistic regression analysis performed to assess influencing factors, the characteristic with the highest level of association with preventive behaviors was concern about MERS infection. This is consistent with the results of previous studies [17,20,21]. Studies that investigated the relationships between perceived risk, anxiety, and preventive behaviors of the public during the H1N1 influenza epidemic found that the more seriously the public perceived MERS symptoms to be, the more likely they were to practice preventive behaviors [17]. A study conducted among nursing students during the 2015 MERS epidemic also indicated that the higher the perceived risk is, the greater the practice of preventive behavior [21]. Female participants, as well as the group that did not support the president or ruling party, were reported to have high practice rates of preventive behaviors. However, additional research is needed to understand why the use of reducing transmission measures had low odds ratios in the group who supported the opposition party. Some observers contend that the government’s attitude in the early stages of the MERS outbreak led to the distrust of the public in MERS response measures [22], resulting in high practice rates of preventive behaviors in the group with low confidence in the government and its response measures. On the other hand, there were no significant differences in rates of preventive behaviors according to major socioeconomic characteristics such as income or education level. However, given that some hierarchy-specific trends in socioeconomic variables were observed, these results might be due to the limited number of study subjects [23].

The fact that concern about MERS infection affects preventive behaviors suggests the benefits of public communication as a means of crisis management in the event of an infectious disease outbreak. In the early days of the MERS outbreak, the government did not specify details regarding scientifically uncertain information in order to reduce public anxiety over the crisis, nor did it disclose which hospitals the confirmed patients had visited. This resulted in increased public distrust in the government [7]. Therefore, knowing that awareness and concern regarding infectious diseases trigger preventive behaviors, it is important to disclose all information on known facts and uncertainties rather than releasing limited information in the hope of reducing public anxiety, so that the government can advise the public to respond with the proper knowledge and appropriate measures.

This study has some limitations. First, the study had a cross-sectional design. It could not reveal causal associations between personal characteristics and preventive behaviors—rather, it could only suggest their relevance. Particularly, it is impossible to state at this time that political affiliation actually led to greater preventive efforts. In addition, changes in preventive behaviors according to changes in the MERS epidemic were not observed, since only one investigation for each specific preventive behavior was conducted. Second, this study could not evaluate the adequacy of preventive behaviors, because it only included questions focusing on whether or not participants practiced these behaviors. It would be useful to evaluate preventive behaviors of respondents qualitatively if questions about the circumstances and frequencies of preventive behavior practice were surveyed in future studies. Finally, bias due to non-response may occur as the characteristics of survey respondents and non-respondents differed. Nevertheless, post-stratification was used to reduce nonresponse bias in surveys, the probability of nonresponse was not equal by characteristics variables within post-strata. However, if the survey period was extended to reduce bias caused by non-response, it is possible that the reaction of the public to crises could change during the course of the investigation [24].

## 5. Conclusions

Despite having a few limitations, this study is meaningful in that it is the first to evaluate preventive behavioral responses of the public during the MERS outbreak in Korea and to present various factors influencing behaviors. The frequency and ease of international travel has created an environment that facilitates the easy spread of infectious diseases between countries. In order to prevent infectious disease epidemics, collective efforts are required on the part of the government and the people. Education on preventive behaviors and appropriate risk communication strategies for the public will be a cornerstone in preventing national infectious disease crises such as the 2015 MERS outbreak.

## Figures and Tables

**Table 1:** General characteristics of the respondents and use of reducing transmission measures (Survey (1)).

Variables		Percentage of Respondents (%) (*n* = 1005)	Percentage of Using Reducing Transmission Measures (%)	
			Wearing a Face Mask (*n* = 155)	Washing Hands More Frequently (*n* = 605)
Gender				
	Male	49.7	11.7	53.4
	Female	50.3	19.2	66.9
Age (years)				
	19–29	17.6	22.5	64.6
	30–39	19.4	19.8	59
	40–49	21.3	13.1	65.4
	50–59	19.7	10.9	53.6
	60–69	14	11	60.2
	≥70	8	14.6	55.5
Occupation				
	Unemployed	8.1	11	53.9
	Farming/fishery	3.5	0	50.6
	Self-employed	15.9	12.6	49.3
	Blue-collar	12.2	12.8	58.5
	White-collar	30	20.1	63.3
	Housewife	22.2	15.2	68.1
	Student	8.1	19.8	61.4
Perceived household economic status				
	Upper	1.7	22.1	53.3
	Upper middle	10.7	17.4	67
	Middle	39.0	18.2	61.1
	Low middle	28.8	11	58.3
	Lower	19.8	15.3	58
Middle East respiratory syndrome coronavirus (MERS-CoV) affected area				
	Non-affected area	49.8	12.6	59.2
	Affected area	50.2	18.3	61.1
Area				
	Non-metropolitan	29.1	9.6	58.1
	Metropolitan	70.9	17.8	61
Presidential approval rating				
	Approval	34.3	11.2	52.1
	Disapproval	54.9	18.7	65.5
	No opinion	10.8	12.3	58.9
Party identification				
	Ruling party	41.4	11.9	57.4
	Opposition party	25.1	19.5	59.7
	No opinion	33.5	16.9	63.9
Concern about MERS-CoV infection				
	Worried	67.3	20	71.8
	Not worried	32.7	6.5	36.2

**Table 2:** Characteristics of the respondents and use of avoidance measures (Survey (2)).

Variables		Percentage of Respondents (%) (*n* = 1004)	Percentage of Using Avoidance Measures (%)			
			Avoiding Outdoor Activities (*n* = 553)	Avoiding Public Transportation (*n* = 409)	Avoiding Healthcare Facilities (*n* = 563)	Avoiding Crowded Places (*n* = 474)
Gender						
	Male	49.8	48.7	33.1	49.6	40.5
	Female	50.2	61.5	48.4	62.5	53.9
Age (years)						
	19–29	17.8	50.6	29.2	58.8	42.9
	30–39	18.9	64.9	50.8	69.6	58.7
	40–49	21.6	58.6	44	61.8	53.6
	50–59	19.4	48.9	35.6	44.9	39.5
	60–69	11.7	48.1	42	45.9	39.9
	≥70	10.7	57.6	43.9	47.6	43
Education						
	Middle school or below	15.6	56.9	47.4	54.4	47.4
	High school	27.5	50.3	36.7	49.8	43
	University	50.2	56.4	40	59.5	47.9
	Graduate school	6.7	62.6	50.7	64.9	60.9
Occupation						
	Unemployed	7.8	47.9	36.8	44.5	33.3
	Farming/fishery	5.0	51.7	48.4	48.3	46.2
	Self-employed	18.7	52.5	33.9	52.6	44.3
	Blue-collar	10.9	49.6	31.7	50.9	42.4
	White-collar	29	58.8	40.3	62	49.9
	Housewife	19.7	63.1	57.7	62.5	57.8
	Student	8.9	45.9	29.3	50.6	39.5
Perceived economic status						
	Upper	2.2	57.9	35.8	57.5	51.4
	Upper middle	8.5	49.9	38.8	56.9	47.4
	Middle	39.4	51.8	38.3	54.5	45.4
	Low middle	27.4	61.8	44.9	60	48.9
	Lower	22.5	55.3	41.7	54.7	48.9
MERS-CoV affected area						
	Non-affected area	49.2	56.5	41.4	55.7	48
	Affected area	50.8	53.8	40.1	56.5	46.4
Area						
	Non-metropolitan	29.3	56.4	42.6	55.2	48
	Metropolitan	70.7	54.6	40	56.5	46.9
Presidential approval rating						
	Approval	32.7	41.6	33.4	40.2	33.4
	Disapproval	58.4	63.2	45.9	65.1	55.4
	No opinion	8.9	52	34.7	55.1	44.1
Party identification						
	Ruling party	39.6	46.4	36.2	44.7	36.3
	Opposition party	29.5	66.2	45.6	68.9	61.8
	No opinion	30.9	55.8	42.1	58.4	47.1
Concern about MERS-CoV infection						
	Worried	52.3	76.3	60	76	68.1
	Not worried	47.7	31.2	19.2	33.8	23.8
Prospects for the control of infection						
	Uncontrolled	26.1	73.2	53.5	73.4	66.9
	Controlled	73.9	49.3	35.8	51.2	41.3

**Table 3:** Association between personal characteristics and nonpharmaceutical preventive behaviors against MERS-CoV.

Variables		Using Reducing Transmission Measures	Using Avoidance Measures
		Odds Ratio (95% CI)	Odds Ratio (95% CI)
Gender			
	Male	Reference	Reference
	Female	1.48 (1.04–2.09)	1.69 (1.11–2.57)
Age (years)			
	19–29	Reference	Reference
	30–39	0.59 (0.33–1.05)	0.71 (0.32–1.57)
	40–49	1.07 (0.6–1.91)	0.48 (0.22–1.02)
	50–59	0.72 (0.39–1.31)	0.39 (0.18–0.86)
	60–69	1.05 (0.54–2.04)	0.43 (0.18–1.03)
	≥70	0.86 (0.39–1.88)	0.45 (0.17–1.19)
Education			
	Middle school or below	- *	Reference
	High school	- *	0.79 (0.42–1.47)
	University	- *	1.31 (0.67–2.57)
	Graduate school	- *	1.73 (0.67–4.46)
Occupation			
	Unemployed	Reference	Reference
	Farming/fishery	1 (0.39–2.54)	0.46 (0.18–1.22)
	Self-employed	0.9 (0.47–1.71)	0.53 (0.25–1.13)
	Blue-collar	1.12 (0.57–2.17)	0.43 (0.19–0.99)
	White-collar	1.4 (0.76–2.59)	0.57 (0.26–1.25)
	Housewife	1.26 (0.66–2.41)	0.71 (0.32–1.59)
	Student	1.41 (0.6–3.3)	0.25 (0.08–0.73)
Perceived economic status			
	Upper	Reference	Reference
	Upper middle	2.24 (0.67–7.49)	0.55 (0.17–1.8)
	Middle	1.53 (0.49–4.8)	0.91 (0.31–2.68)
	Low middle	1.24 (0.39–3.9)	0.91 (0.3–2.75)
	Lower	1.28 (0.4–4.11)	1.04 (0.33–3.26)
MERS-CoV affected area			
	Non-affected area	Reference	Reference
	Affected area	1.03 (0.69–1.54)	1.08 (0.65–1.79)
Area			
	Non-metropolitan	Reference	Reference
	Metropolitan	1.11 (0.76–1.61)	0.85 (0.53–1.37)
Presidential approval rating			
	Approval	Reference	Reference
	Disapproval	1.76 (1.18–2.62)	1.3 (0.82–2.07)
	No opinion	1.03 (0.61–1.75)	1.02 (0.51–2.05)
Party identification			
	Ruling party	Reference	Reference
	Opposition party	0.62 (0.4–0.96)	2.08 (1.22–3.52)
	No opinion	0.8 (0.55–1.18)	1.18 (0.74–1.89)
Concern about MERS-CoV infection			
	Worried	4.47 (3.29–6.07)	9.61 (6.43–14.36)
	Not worried	Reference	Reference
Prospects for the control of infection			
	Uncontrolled	- *	1.39 (0.86–2.26)
	Controlled	- *	Reference

* Not asked in Survey (1).
